# Characterization and phylogenetic analysis of the complete mitochondrial genome of *Pseudothemis zonata* (Odonata: Anisoptera: Libellulidae)

**DOI:** 10.1080/23802359.2020.1839369

**Published:** 2021-01-08

**Authors:** Ying Wang, Yimin Du, Xiang Song, Aijun Huang

**Affiliations:** a School of Life Sciences, Gannan Normal University, Ganzhou, PR China; b National Navel Orange Engineering and Technology Research Center, Ganzhou, PR China

**Keywords:** Libellulidae, mitochondrial genome, *Pseudothemis zonata*, phylogenetic analysis

## Abstract

*Pseudothemis zonata* is a commonly seen dragonfly with a big yellow or white ringlike spot on the third and fourth segments of its abdomen. In this study, we sequenced and analyzed the complete mitochondrial genome (mitogenome) of *P. zonata*. This mitogenome was 15,434 bp long and encoded 13 protein-coding genes (PCGs), 22 transfer RNA genes (tRNAs), and 2 ribosomal RNA unit genes (rRNAs). Gene order was conserved and identical to most other previously sequenced Libellulidae dragonflies. The whole mitogenome exhibited heavy AT nucleotide bias (74.6%). Most PCGs of *P. zonata* have the conventional start codons ATN (six ATG, three ATT, and two ATC), with the exception of *cox1* and *nad1* (TTG). Except for four genes (*cox1*, *cox2*, *cox3*, and *nad5*) end with the incomplete stop codon T−, all other PCGs terminated with the stop codon TAA or TAG. Phylogenetic analysis showed that *P. zonata* got together with *Brachythemis contaminata* with high support value, and the relationships ((*Brachythemis* + *Psolodesmus*) + ((*Hydrobasileus* + *Trigomphus*) + (*Orthetrum* + *Acisoma*))) were supported in Libellulidae.

Insects in the order Odonata are the most ancient invertebrates capable of fight and very diverse, with approximately 6000 species worldwide (Cao and Wu 2019). Libellulidae, a family of over 1000 species, is one of the largest dragonfly family in the world. The libellulids have stout-bodied larvae with the lower lip or labium developed into a mask over the lower part of the face. One of the species in Libellulidae, *Pseudothemis zonata*, is a common dragonfly which is widely distributed in north and east China and in Japan.

Specimens of *P. zonata* were collected from Jingangshan City, Jiangxi Province, China (26°32′N, 114°06′E, June 2018) and were stored in Entomological Museum of Gannan Normal University (Accession number GNU-EPZ03). Total genomic DNA was extracted from tissues using DNeasy DNA Extraction kit (Qiagen, Hilden, Germany). The mitogenome sequence of *P. zonata* was generated using Illumina HiSeq 2500 Sequencing System (Illumina, San Diego, CA). In total, 5.2 G raw reads were obtained, quality-trimmed, and assembled using MITObim version 1.7 (Hahn et al. 2013). By comparison with the homologous sequences of other Libellulidae species from GenBank, the mitogenome of *P. zonata* was annotated using software GENEIOUS R11 (Biomatters Ltd., Auckland, New Zealand).

The complete mitogenome of *P. zonata* is 15,434 bp in length (GenBank accession no. MT371043), and containing the typical set of 13 protein-coding genes (PCGs), 2 ribosomal RNA (rRNA), and 22 transfer RNA (tRNA) genes, and 1 non-coding AT-rich region. Gene order was conserved and identical to most other previously sequenced Libellulidae dragonflies (Tang et al. 2014; Yong et al. 2016; Guan et al. 2019; Wang et al. 2019). The nucleotide composition of the mitogenome was biased toward A and T, with 74.6% of A + T content (A 40.3%, T 34.3%, C 14.5%, G 10.9%). Most PCGs of *P. zonata* have the conventional start codons ATN (six ATG, three ATT, and two ATC), with the exception of *cox1* and *nad1* (TTG). Except for four genes (*cox1*, *cox2*, *cox3*, and *nad5*) end with the incomplete stop codon T−, all other PCGs terminated with the stop codon TAA or TAG. The 22 tRNA genes vary from 63 bp (*trnF* and *trnH*) to 70 bp (*trnK* and *trnV*). Two rRNA genes (*rrnL* and *rrnS*) locate at *trnL1*/*trnV* and *trnV*/control region, respectively. The lengths of *rrnL* and *rrnS* in *P. zonata* are 1283 and 746 bp, with the AT contents of 77.2% and 74.4%, respectively.

Phylogenetic analysis was performed based on the nucleotide sequences of 13 PCGs from 18 Odonata species. Phylogenetic tree was constructed through raxmlGUI version 1.5 (Silvestro and Michalak 2012). Results showed that the new sequenced species *P. zonata* got together with *Brachythemis contaminata* with high support value (Figure 1), indicating genus *Pseudothemis* had a closer relationship with *Brachythemis* within Libellulidae. The relationships ((*Brachythemis* + *Psolodesmus*) + ((*Hydrobasileus* + *Trigomphus*) + (*Orthetrum* + *Acisoma*))) were supported in Libellulidae, and similar results were found in the previous work (Wang et al. 2019). The complete mitochondrial genome of *P. zonata* will provide useful genetic information to study the genetic evolution of Libellulidae dragonflies.

**Figure 1.**
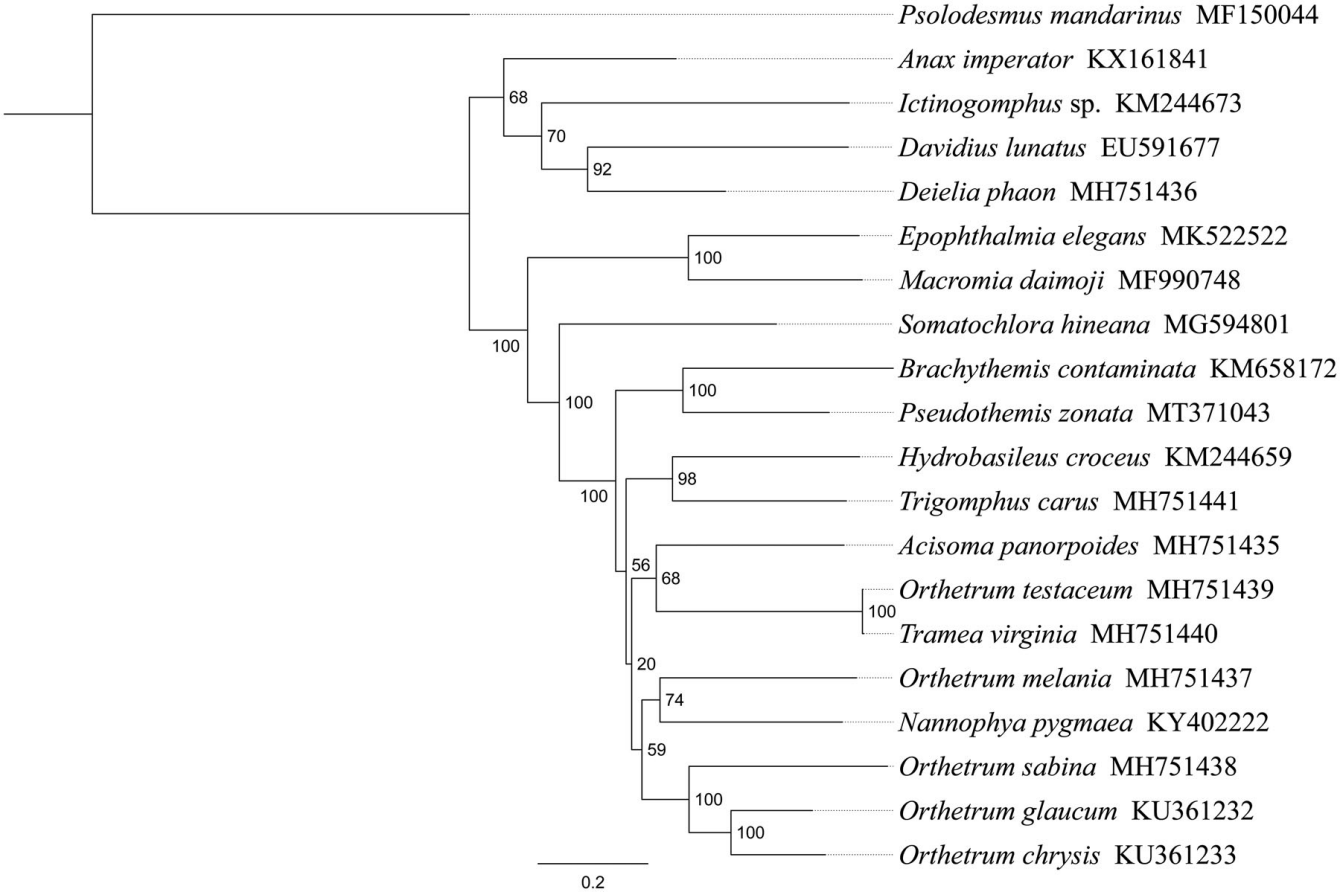
Phylogenetic relationships based on the 13 mitochondrial protein-coding genes sequences inferred from RaxML. Numbers on branches are Bootstrap support values (BS).

## Data Availability

The data that support the findings of this study are openly available in NCBI (National Center for Biotechnology Information) at https://www.ncbi.nlm.nih.gov/, reference number MT371043.
